# Multisource Port Inspection Sensor Fusion with Causal Representation Learning for Cross-Border Anomaly Monitoring

**DOI:** 10.3390/s26134142

**Published:** 2026-07-01

**Authors:** Jiaxin Yin, Zhengjia Lu, Baodi Xiong, Kai Sun, Ruijia Liu, Yachi Liu, Manzhou Li

**Affiliations:** 1 China Agricultural University, Beijing 100083, China; 2 National School of Development, Peking University, Beijing 100871, China; 3 Faculty of Women’s Development, China Women’s University, Beijing 100101, China

**Keywords:** multisource sensor data fusion, cross-border anomaly monitoring, RFID and electronic seal monitoring, cold-chain sensor networks, explainable anomaly detection

## Abstract

With the rapid development of cross-border collaboration, intelligent port construction, and international logistics networks, large volumes of multisource heterogeneous data are continuously generated during cross-border circulation. To address the limitations of traditional financial review and compliance auditing methods in characterizing multisource signal coupling, as well as the tendency of conventional deep models to rely on spurious correlated features with insufficient interpretability, a multisource sensing signal fusion and causally explainable risk identification framework is proposed for cross-border trade anomaly detection. In this framework, electronic trade texts, structured financial declaration fields, GPS/AIS trajectories, port weighing records, RFID data, electronic seal status, X-ray inspection images, cold-chain temperature and humidity records, and vibration data are uniformly modeled as multisource sensing signals in cross-border trade and circulation processes. Subsequently, collaborative representation among textual semantics, attribute fields, logistics status, device records, and entity relationships is achieved through a cross-modal alignment mechanism. On this basis, an engineering-constraint-guided causal risk representation module is designed to reduce the interference of spurious correlated factors, such as regions, ports, transportation modes, and textual styles, in model decisions. Meanwhile, a counterfactual anomaly response module is introduced to analyze the influence of key variable changes on risk outputs, thereby enhancing the model’s ability to identify and explain true anomaly-driving factors. Experimental results show that the proposed method achieves the best overall performance in the cross-border trade anomaly detection task, with Accuracy, Precision, Recall, F1-score, AUC, and PR-AUC reaching 0.927, 0.842, 0.811, 0.826, 0.958, and 0.817, respectively, clearly outperforming baseline models including Logistic Regression, Random Forest, XGBoost, BERT, BERT+MLP, and Multimodal Transformer. In cross-time, cross-region, cross-port, and cross-entity testing scenarios, high F1-score and AUC values are still maintained. Under complex conditions such as text noise, missing modalities, logistics trajectory perturbations, and missing sensing records, only limited performance degradation is observed. Ablation experiments further verify the effective contributions of cross-modal attention, contrastive alignment, causal financial debiasing, counterfactual response, and engineering constraints to performance improvement.

## 1. Introduction

Global economic integration and cross-border e-commerce generate massive volumes of cross-border financial data. This growth drives the need for intelligent financial anomaly monitoring and automated supervision [1]. Supply chain systems continuously generate diverse data throughout the cargo and trade-finance lifecycle. These data span from order placement and export declarations to port inspections, settlement-related document verification, and final delivery. Key data types include invoices, bills of lading, logistics trajectories, and enterprise entity records [2]. Port authorities increasingly adopt electronic systems and intelligent inspection tools. Consequently, supervision transitions from manual reviews to data-driven financial risk monitoring [3].

Fraudulent behaviors, however, grow increasingly complex and concealed. Smugglers use tactics like misinvoicing, tariff classification evasion, and ambiguous commodity descriptions. They also exploit inconsistencies between financial document trails and logistics routes or construct false trade chains [4]. These illicit activities cause tax revenue losses, financial compliance risks, and disrupt market order. Furthermore, they link to severe threats like money laundering, trade-based financial crime, and supply chain security risks [5,6]. Modern cross-border anomalies typically manifest as inconsistencies across multiple data sources rather than single-source errors. Therefore, extracting efficient and explainable financial anomaly signals from heterogeneous data represents a critical challenge for intelligent port supervision.

Traditional risk identification methods rely on manual rules and expert experience. Auditors screen declarations using price thresholds, sensitive country lists, and historical blacklists. These rule-based methods offer high interpretability. However, dynamic changes define the modern cross-border environment. Evaders frequently update their strategies, rendering fixed rules obsolete and causing high false alarm rates [7]. To address this, researchers apply machine learning models like Random Forest and XGBoost to risk prediction. These models automate anomaly detection by analyzing structured fields such as price, weight, and historical statistics [8]. Unfortunately, they struggle to process unstructured texts like commodity descriptions or bill-of-lading remarks. They also fail to model complex relational networks among enterprise entities.

Deep learning offers a promising pathway for cross-border anomaly monitoring [9]. Language models like BERT automatically extract deep semantic representations from document texts, improving the detection of ambiguous declarations [10]. Multimodal learning further fuses text, structured fields, and relational data to uncover complex anomaly patterns [11,12]. Despite these advances, a significant research gap remains in achieving causal robustness and intrinsic interpretability [13]. Current models primarily learn statistical correlations. They mistakenly adopt pseudo-correlated background features, such as specific regions or text styles, as risk indicators. This reliance degrades their generalization across different ports and time periods [14,15]. Furthermore, while post hoc explanation tools like SHAP approximate feature importance after training, they lack formal causal mechanisms. Regulators need models that provide intrinsic, causal-intervention-oriented explainability. Models must natively capture the true drivers of anomalies to support targeted inspections and risk tracing [16]. Finally, practical industrial challenges, including class imbalance, noisy text, and missing modalities, further complicate model deployment [17].

To bridge this gap, we propose a causally explainable multisource fusion framework for cross-border logistics anomaly monitoring. We uniformly model electronic documents, structured fields, and entity relationships as multisource sensing signals. Our approach aligns these diverse signals into a shared feature space. We then introduce an engineering-constraint-guided causal representation mechanism. This mechanism forces the model to ignore spurious correlations and focus on stable risk drivers, such as price deviations or conflicts between commodity descriptions and HS codes. Finally, we design a counterfactual anomaly response strategy. This strategy simulates how modifications to key variables affect the risk output. By doing so, the framework provides intrinsic explainability and directly highlights the root causes of the detected anomalies.

We summarize our primary contributions as follows.

1. We construct a comprehensive multisource framework that integrates document semantics, structured fields, and entity relationships for cross-border anomaly monitoring.
2. We propose a cross-modal alignment mechanism that dynamically fuses diverse signals, establishing explicit interactions among texts, logistical attributes, and enterprise chains.
3. We design an engineering-constraint-guided causal learning module that eliminates spurious environmental correlations and isolates true anomaly-driving representations.
4. We implement a counterfactual response strategy that intervenes on key variables, providing regulators with transparent and traceable evidence for each anomaly decision.

The remainder of this paper is organized as follows. Section 2 reviews related work in cross-border risk identification, multimodal deep learning, and causal inference. Section 3 details the data collection process and the methodology of the proposed causally explainable framework. Section 4 presents the experimental setup, performance comparisons, ablation studies, and robustness evaluations. Finally, Section 5 concludes the paper and discusses future research directions.

## 2. Related Work

### 2.1. Cross-Border Data Anomaly Monitoring and Risk Identification

Early studies on cross-border financial risk identification mainly relied on rule-driven mechanisms, in which expert knowledge was converted into fixed risk rules and anomalous samples were screened through rule matching [1]. For example, when the declared price is substantially lower than the historical average, when the commodity name is inconsistent with the HS code, or when a trading entity appears on a historical blacklist, the corresponding sample is labeled as high risk [5]. From the perspective of engineering-oriented supervision, such methods can be regarded as prior-knowledge-based anomaly-state discrimination systems, with advantages such as transparent rules, strong interpretability, and easy integration into existing port supervision workflows [18]. However, cross-border logistics chains are long, the involved entities are numerous, and declaration strategies and evasion methods change rapidly. Therefore, fixed rules are difficult to maintain sufficient coverage of dynamically evolving anomaly patterns [19]. In addition, rule-based systems usually require substantial manual maintenance. When complex scenarios involve joint anomalies in document information, logistics states, and entity relationships, insufficient coverage and high missed-detection rates may easily occur. Subsequently, statistical learning and traditional machine learning methods were introduced into cross-border risk analysis, with the core idea of learning statistical relationships between declaration variables and financial risk labels from historical samples [20]. For example, Logistic Regression models risk probability through a linear function, whereas Random Forest and XGBoost characterize nonlinear feature relationships through tree structures and ensemble learning. These methods can use structured fields, such as price, quantity, weight, country and HS code, for automated modeling, thereby providing stronger data adaptability than manual rules. However, traditional machine learning methods still mainly rely on structured fields and manually designed features, making it difficult to fully exploit unstructured anomalous clues contained in invoice descriptions, contract remarks, bill-of-lading notes, and customs declaration texts [21]. Meanwhile, these methods usually treat each declaration sample as an independent instance, which makes it difficult to characterize collaborative relationships, historical cooperation links, and group-level anomaly patterns among enterprises. Therefore, their applicability remains limited in real cross-border engineering supervision scenarios. To overcome the limitation of treating declarations as isolated events, graph-based customs fraud detection methods have recently emerged. By modeling enterprises, ports, and logistics agents as nodes, and historical trade interactions as edges, graph neural networks can capture topological anomalies and collusive fraud behaviors that traditional tabular models often miss. However, these graph-based methods usually focus on homogeneous relationship structures and struggle to align complex unstructured document semantics with node attributes.

### 2.2. Multisource Sensing Signal Fusion and Multimodal Deep Learning

In recent years, deep learning has achieved remarkable progress in natural language processing, anomaly detection, and multisource data fusion, providing a new technical pathway for cross-border trade finance anomaly monitoring [22]. Deep learning methods can automatically learn high-dimensional feature representations through multilayer nonlinear mappings, thereby reducing the dependence on manual feature engineering [9]. For instance, TextCNN extracts local semantic patterns from texts through convolutional operations, BiLSTM captures contextual dependencies through bidirectional sequential modeling, and BERT obtains deep semantic representations through the Transformer architecture and large-scale pretraining [23]. These methods can directly extract implicit anomaly features from document texts, such as commodity descriptions, bill-of-lading notes, and contract summaries, thereby enhancing the ability of models to understand ambiguous declarations, abnormal expressions, and potential risk semantics [24]. With the development of multimodal learning, texts, structured fields, and relational data have begun to be jointly modeled to improve anomaly identification in complex scenarios [25]. In cross-border supervision, electronic documents, declaration fields, logistics states, and enterprise relationships can be regarded as sensing signals from different sources. The key to fusion modeling lies in mapping heterogeneous data into a unified feature space and achieving information fusion through attention mechanisms or cross-modal interaction structures [26]. Similar ideas have been applied to financial risk control, bill auditing, and supply chain analysis, enabling models to jointly use textual semantics and structured variables for risk prediction. Although multimodal deep learning has shown strong potential, several challenges remain in cross-border engineering monitoring scenarios [27]. First, texts often involve mixed Chinese and English expressions, dense professional terminology, frequent abbreviations, and nonstandard writing, resulting in noisy textual signals. Second, obvious asynchrony and semantic alignment difficulties exist among different modalities. Third, multimodal fusion models usually contain a large number of parameters and depend heavily on data quality and sample scale, whereas real trade anomaly samples are often limited and highly imbalanced [21]. In addition, most existing multimodal models are correlation-driven methods that mainly learn statistical associations between input features and risk labels. Due to insufficient characterization of anomaly formation mechanisms and engineering constraint relationships, these models are easily affected by pseudo-correlated features [28].

### 2.3. Causal Inference and Explainable Anomaly Identification

To alleviate the generalization limitations caused by correlation-based learning, causal inference and causal representation learning have attracted increasing attention in recent years. Unlike traditional correlation modeling, causal inference focuses more on whether changes in variables truly lead to changes in outcomes [18]. Its theoretical foundation is usually built on structural causal models and intervention analysis frameworks, through which stable financial risk-driving factors are identified by analyzing causal pathways among variables [29]. In risk prediction and anomaly detection tasks, causal learning helps distinguish true risk factors from pseudo-correlated background features, thereby improving model robustness under distribution shifts [30]. To address performance degradation caused by environment-specific biases, domain-adversarial neural networks have been widely applied to extract features that are indistinguishable across different domains, thereby promoting generalization. Building upon this, invariant risk minimization introduces a regularization mechanism that enforces the optimal classifier to be the same across all training environments, explicitly driving the model to abandon spurious correlations. Furthermore, recent causal domain-generalization methods integrate structural causal models with representation learning to isolate causal mechanisms from environmental confounders, enabling robust predictions even under severe out-of-distribution scenarios [31]. For example, causal representation learning can constrain models to learn environment-invariant features, so that anomaly discrimination remains stable across different environments. Counterfactual inference constructs contrastive samples by asking how model outputs would change if a key variable were modified, thereby analyzing the true influence of different features on prediction results [32]. However, existing causal learning studies are mostly concentrated on structured data or single-modal tasks, while insufficient attention has been paid to multisource heterogeneous sensing signals in cross-border finance-related scenarios, such as document semantics, declaration fields, logistics states, and entity relationships [33]. Meanwhile, most methods focus more on causal inference itself, and causal constraints are rarely integrated with multimodal deep feature learning within a unified engineering monitoring framework. As a result, it remains difficult for models to simultaneously achieve semantic representation capability, cross-modal fusion capability, and causal robustness [34]. In addition, existing methods still have limitations in interpretability. Although many models can output risk probabilities, they fail to clearly explain which document fields, logistics states, or entity relationships jointly trigger anomalies. This remains inconsistent with the requirements of intelligent port supervision for risk tracing, targeted inspection, and decision support [35].

## 3. Materials and Method

**Figure 1:**
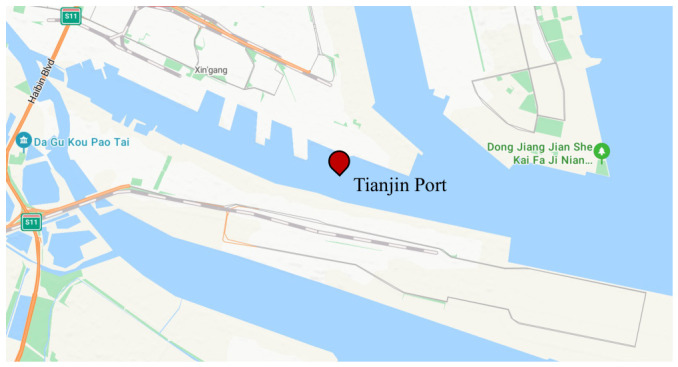
Geographical location of Tianjin Port.

A multisource dataset was constructed for cross-border logistics and financial anomaly monitoring, with the data collection period spanning from January 2022 to December 2024. The data were mainly obtained from Tianjin Port and its surrounding freight forwarding enterprises, the cross-border service platform of Tianjin Dongjiang Comprehensive Bonded Zone, selected logistics partners associated with Ningbo Zhoushan Port, agricultural product export and cold-chain transportation enterprises in Yantai, Shandong Province, as well as public statistics and publicly available vessel trajectory data, as summarized in Table 1. Tianjin Port was selected as the primary research context because it is a major coastal port in northern China and serves as an important maritime gateway for the Beijing–Tianjin–Hebei region. Its strategic location in the Bohai Sea region, intensive cross-border logistics activities, and diverse multimodal transportation scenarios make it suitable for studying logistics anomaly monitoring. The geographical location of Tianjin Port is shown in Figure 1.

Electronic document data were mainly collected from historical desensitized document records provided by freight forwarding enterprises around Tianjin Port, customs declaration agencies, and cooperating enterprises in Tianjin Dongjiang Comprehensive Bonded Zone. A portion of agricultural product export documents was obtained from agricultural export enterprises and cold-chain logistics enterprises in Yantai, Shandong Province. The data include commercial invoices, packing lists, bills of lading, customs declarations, contract summaries, and commodity description texts. These documents were submitted by import and export enterprises during the declaration process and record information such as commodity names, specifications, usage descriptions, packaging methods, transportation descriptions, and consignee or consignor information. For electronic-form data, fields were directly parsed according to predefined templates. For PDF documents or scanned documents, text extraction was performed using high-resolution scanning devices and OCR tools.

Structured declaration data were mainly obtained from desensitized declaration records in the systems of cooperating customs declaration enterprises, cross-border e-commerce service enterprises, and comprehensive bonded zones. Public data from China Customs statistics and UN Comtrade were further incorporated to construct reference intervals for similar commodity prices and background information. The structured fields include declared price, quantity, weight, currency, country of origin, destination country, HS code, transportation mode, transaction mode, declaration time, and historical declaration statistics of enterprises. Such data are usually entered into systems after online declaration by enterprises and are characterized by clearly defined fields, strong constraints, and complete timestamps. During data collection, currencies, weight units, and quantity units were uniformly converted, while missing values, outliers, and duplicate declaration records were marked. These structured fields also provide direct evidence for trade finance risk assessment.

Logistics trajectory data were mainly collected from transportation node records provided by logistics partners associated with Tianjin Port and Ningbo Zhoushan Port, GPS vehicle-mounted positioning terminals used by cold-chain transportation enterprises, AIS ship automatic identification systems, and publicly available AIS vessel trajectory data. For land transportation samples, longitude, latitude, speed, direction, positioning time, stopping status, and transportation node information recorded by vehicle GPS terminals were primarily collected. For maritime transportation samples, ship position, sailing speed, heading, port calls, and route variation information recorded by AISs were collected. To enhance experimental reproducibility, public AIS trajectory data and selected public GPS trajectory data were also used for auxiliary validation of the trajectory anomaly detection and route deviation analysis modules. Specifically, out of the 42,680 GPS land trajectories, 34,144 samples, accounting for 80%, are proprietary operational data collected from logistics partners, while 8536 samples, accounting for 20%, are public trajectories. For the 31,900 AIS maritime trajectories, 19,140 samples, accounting for 60%, represent proprietary data from associated shipping enterprises, and the remaining 12,760 samples, accounting for 40%, are derived from public maritime databases.

Port inspection data were mainly obtained from on-site supervision records of port logistics parks, checkpoint systems in comprehensive bonded zones, and cooperating logistics enterprises. Specifically, these data include weighbridge records, channel camera records, license plate recognition records, RFID identification records, electronic seal status records, and partial X-ray inspection images collected from logistics parks around Tianjin Port, Tianjin Dongjiang Comprehensive Bonded Zone, and cooperative scenarios associated with Ningbo Zhoushan Port. Weighing data were automatically recorded by port weighbridges or dynamic weighing devices and compared with declared weights. X-ray images were collected by container or parcel inspection equipment to assist in determining whether cargo shape, loading density, and declared content were consistent. Camera data mainly recorded vehicle entry and exit, container appearance, and inspection procedures. RFID and electronic seal data were used to record the identification status and opening or closing status of containers or cargo batches at key nodes.

Environmental status data were mainly obtained from transportation monitoring devices deployed on agricultural product export cold-chain routes in Yantai, Shandong Province, as well as from cold-chain warehousing enterprises, bonded warehouses, and selected cross-border e-commerce logistics enterprises. The data mainly include temperature, humidity, vibration, door-opening status, and recording time. For agricultural products, food, pharmaceuticals, and other goods sensitive to transportation environments, such data can reflect environmental stability and cargo preservation status during transportation. During collection, temperature and humidity sensors automatically recorded environmental changes at fixed time intervals, electronic seals or magnetic door sensors recorded container opening and closing status, and vibration sensors recorded abnormal shocks during transportation. To ensure the scientific validity and reproducibility of the study, a rigorous anomaly labeling protocol was established to explicitly define the origin of the anomaly risk labels. These labels were systematically derived from two primary sources, directly addressing the spectrum from confirmed fraud cases to high-risk inspection findings. First, strong positive labels were extracted directly from confirmed customs penalty records and official violation logs provided by cooperating port authorities and customs declaration agencies. These records represent explicitly verified illicit activities, such as confirmed smuggling, severe under-invoicing, and tariff classification evasion, which resulted in formal administrative or legal penalties. Second, weak labels were generated from high-confidence inspection suspicions logged during frontline port operations, combined with expert assessments. These operational indicators include unresolved discrepancies in weighbridge records, abnormal density patterns flagged by X-ray operators, and broken electronic seal alerts. To convert these operational suspicions into reliable ground-truth labels, a multi-stage annotation process was conducted following a double-blind protocol. Three senior customs auditors and domain experts independently reviewed the historical declarations, associated documents, and sensor records to assign the final risk labels. In cases of disagreement, a consensus was reached through joint discussion, ensuring that general risk indicators were rigorously verified rather than automatically labeled as anomalies. Among the total 86,320 declaration samples, 7520 were ultimately identified as positive anomaly samples through this rigorous process, while the remaining 78,800 were classified as negative normal samples, resulting in a class imbalance ratio of approximately 1 to 10.5. To quantify the inter-annotator agreement during the evaluation of suspected cases, the Cohen Kappa coefficient was calculated, yielding an average value of 0.86, which indicates a high level of consistency and reliability in the generated labels.

To ensure the legitimacy, compliance, and ethical integrity of the data pipeline, stringent data governance protocols were enforced throughout the collection process. Formal data-sharing agreements and non-disclosure agreements were established with all collaborating entities, including port authorities, customs agencies, and logistics enterprises, granting explicit legal permissions for academic research use. Ethical approval was obtained from the institutional review board of the affiliated research institution prior to data acquisition. To strictly protect commercial confidentiality and individual privacy, a rigorous anonymization procedure was implemented before any data was transferred to the research servers. Specifically, sensitive identifiable information, such as enterprise names, contact details, personal identification numbers, and exact financial account information, was irreversibly masked using one-way cryptographic hashing algorithms. Furthermore, continuous variables such as exact cargo values were subjected to distribution-preserving noise injection to prevent reverse engineering, ensuring that the dataset retains its structural properties for machine learning while fully complying with relevant data privacy regulations. This governance process also supports secure research use of financial compliance-related records.

### 3.1. Data Preprocessing and Augmentation Strategy

In the task of cross-border document fraud detection, raw data usually exhibit pronounced heterogeneity, noise, and distributional imbalance. Therefore, before model training, multimodal data need to be normalized and distributionally optimized through systematic data preprocessing and data augmentation strategies. In essence, data preprocessing aims to map the original observations *X* into a representation space $\tilde{X}$ that is more suitable for model learning through a series of deterministic or statistical transformations, namely by constructing a mapping function $f_{\mathrm{pre}}:X\to \tilde{X}$, thereby improving feature separability and stability. Data augmentation expands the sample distribution by introducing a perturbation function $f_{\mathrm{aug}}$ while preserving label semantics, so as to improve model robustness to input perturbations. This process can essentially be represented as the construction of an extended distribution $p^{\prime }\left(x\right)$ based on the original data distribution p(x).

For textual modality preprocessing, the core principle is to make textual representations more stable and comparable through normalization and semantic alignment of unstructured texts. Let the original text sequence be $x=\{w_{1},w_{2},\ldots ,w_{n}\}$. A normalized sequence $\tilde{x}$ is obtained through the preprocessing function $f_{\mathrm{text}}$, namely $\tilde{x}=f_{\mathrm{text}}\left(x\right)$. In this process, abnormal characters and invalid symbols are first removed by a rule-based function $f_{\mathrm{clean}}$, which can be represented as a filtering mapping over the character space V. Subsequently, synonymous expressions are mapped into a unified vocabulary space through a terminology normalization function $f_{\mathrm{norm}}$, for example, by mapping expressions such as “pcs” and “pieces” to a unified identifier, thereby reducing lexical sparsity. Furthermore, different measurement expressions are converted into a unified dimension through a unit normalization function $f_{\mathrm{unit}}$, so that the numerical semantics embedded in the text become consistent. Finally, the text sequence is mapped into a vector representation $h_{\mathrm{text}}$ through the embedding function ϕ(·):

(1) $$h_{\mathrm{text}}=\phi \left(\tilde{x}\right)=\phi \left(f_{\mathrm{unit}}\left(f_{\mathrm{norm}}\left(f_{\mathrm{clean}}\left(x\right)\right)\right)\right).$$

This process essentially reduces noise interference and expression variation in the textual modality, enabling the model to focus more on semantic-level risk signals. In cross-border scenarios, where mixed Chinese–English expressions and frequent abbreviations are common, this preprocessing procedure is particularly important for reducing semantic ambiguity and improving representational consistency. For structured data preprocessing, the core objective is to transform variables of different scales and types into a learnable space through numerical standardization and categorical encoding. For a continuous variable $x_{i}$, normalization or standardization is applied. For example, the standardization operation can be expressed as follows:

(2) $${\tilde{x}}_{i}=\frac{x_{i}-\mu_{i}}{\sigma_{i}},$$

where $\mu_{i}$ and $\sigma_{i}$ denote the mean and standard deviation of variable $x_{i}$ in the training set, respectively. This operation can eliminate the adverse influence of scale differences among variables on model training. For a categorical variable $c_{j}$, an embedding function e(·) is used to map it into a low-dimensional vector representation $h_{j}=e\left(c_{j}\right)$, so that discrete attributes can participate in feature learning within a continuous space. Meanwhile, for missing values, a conditional expectation imputation strategy is introduced, in which missing variables are estimated using other observed feature information. This can be expressed as:

(3) $$x_{\mathrm{miss}}=E\left[x|X_{\mathrm{obs}}\right].$$

In addition, because different currencies and measurement units are involved in cross-border data, a conversion function $f_{\mathrm{conv}}$ is required to unify prices and quantities into standard scales. For example:

(4) $$x_{\mathrm{price}}^{\mathrm{USD}}=r_{\mathrm{currency}}\cdot x_{\mathrm{price}},$$

where $r_{\mathrm{currency}}$ denotes the exchange-rate conversion factor. Through the above processing steps, scale inconsistency and abnormal interference in structured data can be effectively reduced. For relational modality construction, the core idea is to characterize potential dependencies among samples by introducing graph structures or relational vectors, thereby supplementing group-level behavioral information that is missing from individual samples. Let the enterprise set be denoted as V, and let the relationships among enterprises form a graph G=(V,E), where the edge weight $A_{ij}$ represents the association strength between enterprise *i* and enterprise *j*, such as co-occurrence frequency or transaction frequency. Based on this graph structure, node representations can be extracted through graph representation learning:

(5) $$h_{i}^{\left(l+1\right)}=\sigma \left(\sum_{j\in N(i)}A_{ij}W^{\left(l\right)}h_{j}^{\left(l\right)}\right),$$

where $h_{i}^{\left(l\right)}$ denotes the node representation at the *l*-th layer, $W^{\left(l\right)}$ is a learnable parameter, and σ is a nonlinear activation function. This propagation mechanism aggregates information from neighboring nodes into the current node, thereby capturing collaborative risk patterns among enterprises, such as anomalous chains or group-based behaviors. By introducing the relational modality, the model can be extended from individual risk modeling to group risk modeling, significantly improving its ability to identify complex fraud behaviors. For data augmentation, the theoretical basis lies in constructing perturbed samples in the input space so that the model can learn a more stable decision boundary. For the textual modality, a semantics-preserving perturbation function $f_{\mathrm{aug}}^{\mathrm{text}}$ can be defined, such as synonym replacement or expression-variant generation, so that the augmented sample $\tilde{x}$ satisfies the semantic consistency constraint:

(6) $$y\left(\tilde{x}\right)=y\left(x\right),\;\tilde{x}=f_{\mathrm{aug}}^{\mathrm{text}}\left(x\right),$$

where y(·) denotes the labeling function. This strategy can improve model robustness to expression variations. In the structured modality, augmentation can be achieved by introducing small random perturbations to non-critical variables, such as:

(7) $${\tilde{x}}_{i}=x_{i}+\epsilon ,\;\epsilon ∼N\left(0,\sigma^{2}\right).$$

This method expands the data distribution without changing the sample semantics. In addition, a resampling strategy can be adopted to alleviate class imbalance, making the distribution of positive and negative samples more balanced. Within the causal learning framework, data augmentation is further extended to counterfactual sample generation. The core idea is to construct contrastive samples that differ from the original sample in certain variables while keeping other conditions unchanged. Let the original sample be *x* and the key variable be $x_{k}$. Then, the counterfactual sample $x^{cf}$ can be expressed as:

(8) $$x^{cf}=\left(x_{1},\ldots ,x_{k}^{\prime },\ldots ,x_{n}\right),$$

where $x_{k}^{\prime }$ denotes the intervened value of the key variable. By comparing the model outputs on *x* and $x^{cf}$, the causal effect of variable $x_{k}$ on the prediction result can be characterized as follows:

(9) $$\Delta y=f\left(x\right)-f\left(x^{cf}\right).$$

This mechanism not only enhances model sensitivity to changes in key variables, but also provides a basis for subsequent explainability analysis. By incorporating counterfactual samples into the training process, the model can be guided to learn more stable causal features, thereby reducing its dependence on pseudo-correlated signals.

### 3.2. Proposed Method

#### 3.2.1. Overall Architecture

We propose a multisource fusion and causally explainable framework for cross-border logistics anomaly monitoring. Unlike conventional methods relying solely on isolated data types, our model jointly perceives textual semantics, structured attributes, and entity relationships to identify risks. The end-to-end pipeline consists of four main stages. First, specialized encoders process three core inputs: a pretrained language model extracts the semantic representation $h_{i}^{t}$ from document texts $x_{i}^{t}$; a tabular encoder processes continuous and categorical declaration fields $x_{i}^{s}$ into a structured representation $h_{i}^{s}$; and a graph-based encoder models enterprise associations $x_{i}^{r}$ to output a relational representation $h_{i}^{r}$. Second, a cross-modal attention mechanism aligns and interacts these representations in a unified latent space, generating a comprehensive multisource fused representation $z_{i}$. Third, an engineering-constraint-guided causal debiasing module filters $z_{i}$ to eliminate environmentally spurious correlations (e.g., regional attributes or textual styles). This isolates true anomaly-driving factors, yielding a stable, debiased risk representation $z_{i}^{c}$. Finally, a counterfactual anomaly response module simulates interventions on key variables (e.g., correcting HS codes or standardizing prices). By comparing the risk outputs of the original and counterfactual samples, the model enhances its sensitivity to true risk drivers. The debiased representation is then fed into a classification layer to output the final anomaly risk score alongside explainable risk attributions.

#### 3.2.2. Multisource Sensing Representation and Cross-Modal Alignment Module

In the multisource sensing representation and cross-modal alignment module, each preprocessed sample is represented as $x_{i}=\left\{x_{i}^{t},x_{i}^{s},x_{i}^{r}\right\}$, where $x_{i}^{t}$ denotes the electronic document text sequence, $x_{i}^{s}$ denotes structured declaration and logistics state fields, and $x_{i}^{r}$ denotes entity relationship features. For the textual modality, a pretrained language model is adopted as the semantic encoder. Specifically, the BERT-base model is employed to extract deep semantic features. Texts such as invoice commodity descriptions, packing-list descriptions, bill-of-lading notes, customs declaration remarks, and contract summaries are first fed into the token embedding layer and then encoded by multilayer Transformer blocks to obtain contextual semantic representations. Let the embedding of the text sequence be $E_{i}^{t}=\left\{e_{i,1}^{t},\cdots ,e_{i,L}^{t}\right\}$. The text encoding process can then be expressed as $H_{i}^{t}={\mathrm{Transformer}}_{t}\left(E_{i}^{t};\theta_{t}\right)$, and the sample-level textual vector is obtained through a special-token representation or attention pooling as $h_{i}^{t}=\mathrm{Pool}\left(H_{i}^{t}\right)$, outputting a 768-dimensional textual feature vector. This design can capture contextual dependencies among commodity names, materials, specifications, uses, packaging methods, and transportation descriptions, thereby avoiding semantic omissions caused by reliance on keyword matching alone.

**Figure 2:**
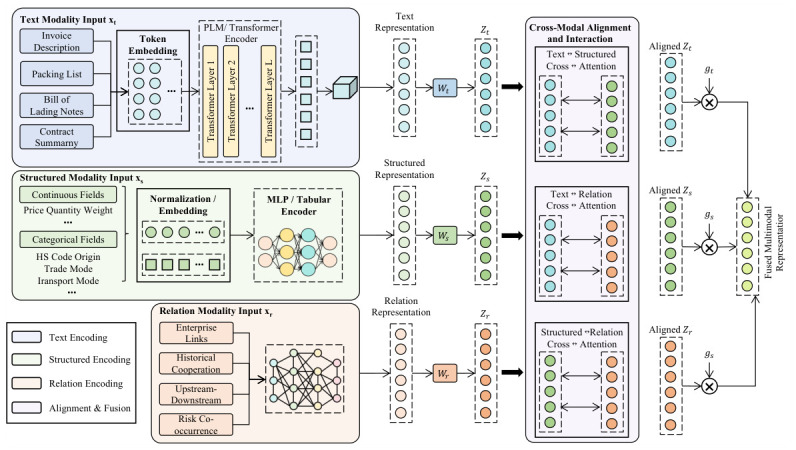
The multisource sensing representation and cross-modal alignment module fuses document texts, declaration fields, and entity relationships into a unified risk representation.

For the structured modality, continuous fields are first normalized and then fed into a linear mapping layer, while categorical fields such as HS code, country of origin, transportation mode, and port node type are converted into dense vectors through embedding matrices. As shown in Figure 2, let the continuous variables be $u_{i}$ and the categorical variables be $v_{i}$. The structured representation can be written as $h_{i}^{s}={\mathrm{MLP}}_{s}\left(\left[W_{u}u_{i};\mathrm{Emb}\left(v_{i}\right)\right];\theta_{s}\right)$, where [·;·] denotes the concatenation operation. The ${\mathrm{MLP}}_{s}$ consists of several linear layers, nonlinear activations, normalization layers, and dropout layers, and is used to model combinational relationships among price, quantity, weight, currency, HS code, transportation mode, and inspection state. The output dimension of this structured representation $h_{i}^{s}$ is set to 256. For the relational modality, if entity associations are represented as a graph, enterprise entities are treated as nodes, while historical transactions, consignee–consignor relationships, or customs-declaration agency relationships are treated as edges; a GraphSAGE (Graph Sample and Aggregate) encoder is then used to obtain the relationship representation of the current declaration entity. If relationship information is provided as statistical features, it is mapped by a relationship feature encoding network. This process can be uniformly expressed as $h_{i}^{r}={\mathrm{Encoder}}_{r}\left(x_{i}^{r};\theta_{r}\right)$, which generates a 256-dimensional relationship feature vector. This vector is used to characterize link risk information such as enterprise historical cooperation frequency, upstream and downstream stability, risk co-occurrence degree, and abnormality of associated entities. Because multisource sensing signals differ substantially in acquisition devices, data forms, semantic granularity, and temporal distributions, direct concatenation may cause a dominant modality to control model decisions, or may prevent explicit associations from being established among document semantics, declaration fields, and logistics states. Therefore, a cross-modal projection and alignment mechanism is further designed to map $h_{i}^{t}$, $h_{i}^{s}$, and $h_{i}^{r}$ into the same latent space, yielding $z_{i}^{t}=W_{t}h_{i}^{t}+b_{t}$, $z_{i}^{s}=W_{s}h_{i}^{s}+b_{s}$, and $z_{i}^{r}=W_{r}h_{i}^{r}+b_{r}$. After dimensional unification, cross-modal attention is adopted to achieve interactions among modalities. Taking the attention of the document textual representation to structured declaration fields as an example, the attention process can be expressed as $A_{t\leftarrow s}=\mathrm{softmax}\left(Q_{t}K_{s}^{⊤}/\sqrt{d}\right)V_{s}$, where $Q_{t}=z_{i}^{t}W_{Q}$, $K_{s}=z_{i}^{s}W_{K}$, $V_{s}=z_{i}^{s}W_{V}$, and *d* denotes the latent-space dimension, which is uniformly set to 256 for all modalities to ensure consistent cross-modal interactions. Similarly, bidirectional attention can be constructed between text and relationships as well as between structured fields and relationships, enabling commodity descriptions to actively align with HS codes, price intervals, weight states, and entity relationship features, while allowing relationship-chain information to reversely adjust the understanding of current declaration fields. The interaction representations from different directions are then fed into a gated fusion unit to obtain the final multimodal fused representation:

(10) $$z_{i}=g_{t}⊙{\tilde{z}}_{i}^{t}+g_{s}⊙{\tilde{z}}_{i}^{s}+g_{r}⊙{\tilde{z}}_{i}^{r},$$

where $g_{m}=\sigma \left(W_{g}\left[{\tilde{z}}_{i}^{t};{\tilde{z}}_{i}^{s};{\tilde{z}}_{i}^{r}\right]+b_{g}\right)$ denotes the modality weight, and ⊙ denotes element-wise multiplication. This gating mechanism can dynamically adjust the contribution of each modality according to the signal quality and anomaly source of different samples. For instance, when document descriptions are ambiguous but declared prices or weights are clearly abnormal, the weight of the structured modality is increased. When entity relationship anomalies are prominent, the influence of the relational modality on the final representation is enhanced. When obvious conflicts occur between document semantics and HS codes, the interaction weights between textual and structured fields are amplified. To further ensure discriminative consistency of aligned representations, a cross-modal contrastive constraint is introduced, so that representations of different sensing signals from the same sample are pulled closer in the latent space, while modality representations from different samples remain distinguishable. Taking the textual and structured modalities as an example, the alignment loss can be expressed as:

(11) $$L_{ts}=-\log\frac{\exp(\mathrm{sim}\left(z_{i}^{t},z_{i}^{s}\right)/\tau )}{\sum_{j}\exp\left(\mathrm{sim}\left(z_{i}^{t},z_{j}^{s}\right)/\tau \right)},$$

where sim(·) denotes the similarity function, and τ is the temperature coefficient. Similar constraints can also be constructed between the relational modality and the textual and structured modalities, resulting in the final alignment objective:

(12) $$L_{align}=L_{ts}+L_{tr}+L_{sr}.$$

Through this design, anomaly cues from document texts, declaration fields, and entity relationships can be learned separately, while semantic consistency and complementarity among different sensing signals can also be explicitly strengthened. For cross-border logistics anomaly monitoring, this structure facilitates the identification of complex anomaly patterns, such as inconsistencies between commodity descriptions and HS codes, mismatches between declared prices and commodity semantics, inconsistencies between weight records and commodity attributes, deviations between logistics links and declared routes, and abnormal coupling between entity historical relationships and current transaction behaviors. Accordingly, the problems of insufficient information from a single data source and semantic fragmentation across modalities can be alleviated, providing a more stable and complete risk representation foundation for subsequent causal debiasing and counterfactual anomaly response.

#### 3.2.3. Engineering-Constraint-Guided Causal Risk Representation Debiasing Module

The engineering-constraint-guided causal risk representation debiasing module receives the fused representation $z_{i}\in R^{C}$ obtained from the previous stage, where *C* denotes the number of fused feature channels. The objective of this module is not merely to improve classification accuracy on the training set, but to extract risk representations from multisource sensing signals that remain stable across different times, regions, ports, and entity environments.

**Figure 3:**
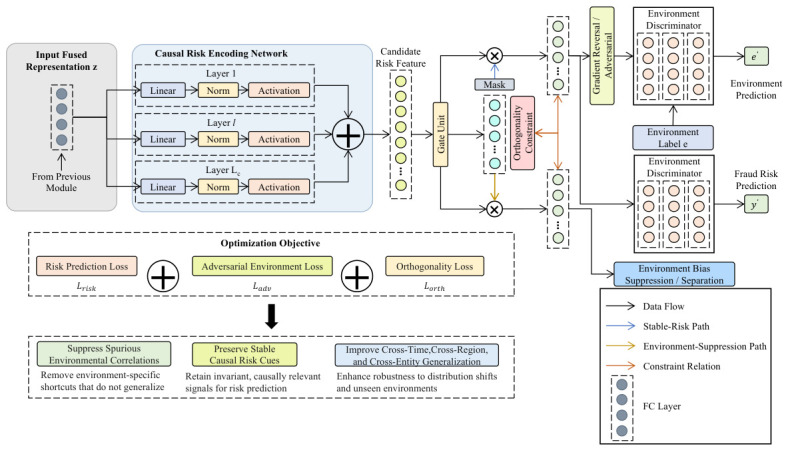
The engineering-constraint-guided causal risk representation debiasing module separates stable risk features from environmental bias through causal gating, adversarial environment learning, and orthogonality constraints.

As shown in Figure 3, the fused representation is first passed through an $L_{c}$-layer causal risk encoding network. In our specific implementation, the number of encoding layers is set to $L_{c}=3$. The initial input dimension $C_{0}$ aligns with the multimodal fused dimension of 256, and the subsequent hidden dimensions are uniformly configured as 256 to maintain information capacity. Each layer consists of a linear mapping, normalization, nonlinear activation, and residual connection. The input width of the *l*-th layer is $C_{l}$, the output width is $C_{l+1}$, the height can be interpreted as the sample dimension *B*, and the number of channels corresponds to the hidden feature dimension $C_{l}$. This process is expressed as:

(13) $$q_{i}^{l+1}=\phi \left(\mathrm{Norm}\left(W_{c}^{l}q_{i}^{l}+b_{c}^{l}\right)\right)+R_{l}q_{i}^{l},\;q_{i}^{0}=z_{i},$$

where $W_{c}^{l}\in R^{C_{l+1}\times C_{l}}$, $b_{c}^{l}\in R^{C_{l+1}}$, $R_{l}$ is the residual mapping, and ϕ(·) is a nonlinear function. For reproducible implementation, the normalization is realized via Layer Normalization, and the activation function employs the Gaussian Error Linear Unit. A dropout operation with a probability of 0.1 is also applied within each block to prevent overfitting. After $L_{c}$ layers of encoding, the candidate risk representation $q_{i}$ is obtained. Subsequently, a causal gating layer is introduced to decompose $q_{i}$ into the stable risk component $q_{i}^{risk}$ and the environmental bias component $q_{i}^{env}$:

(14) $$m_{i}=\sigma \left(W_{m}q_{i}+b_{m}\right),\;q_{i}^{risk}=m_{i}⊙q_{i},\;q_{i}^{env}=\left(1-m_{i}\right)⊙q_{i},$$

where $m_{i}$ is a learnable causal mask, $W_{m}\in R^{C_{q}\times C_{q}}$, and $C_{q}$ denotes the number of channels after causal encoding. To rigorously justify this decomposition from the perspective of a structural causal model, we conceptualize the generation of the observed multimodal representation $q_{i}$ as a function of two independent latent subspaces: the true causal factors *C* that universally determine the anomaly outcome *Y*, and the environmental confounders *E* that spuriously correlate with *Y* due to selection bias across different ports or operational regions. According to the independent causal mechanisms principle, the joint distribution can be factorized, and the causal mechanism P(Y|C) remains invariant under interventions on *E*. In this context, the mask $m_{i}$ functions not merely as a deterministic feature selector, but as a differentiable approximation of the structural assignment equations $C\leftarrow f_{C}\left(q_{i}\right)$ and $E\leftarrow f_{E}\left(q_{i}\right)$. By continuously updating $m_{i}$ through the subsequent adversarial environment prediction objective, the network effectively simulates a soft intervention do(E), enforcing the separation of invariant causal pathways from shifting environmental contexts. Consequently, $m_{i}$ isolates the stable structural parents of the anomaly risk, ensuring that $q_{i}^{risk}$ captures only the genuine anomaly-driving factors, aligning directly with the confounder disentanglement paradigm in formal causal inference. This design enables the model to automatically select feature components that are more stably related to anomaly risk and suppress information that may carry environmental bias. The environmental variables may represent non-core background factors such as regions, ports, transportation modes, time windows, or enterprise categories. To further reduce the environmental identifiability of $q_{i}^{risk}$, an environmental discriminator composed of $L_{d}$ fully connected layers is introduced. Specifically, the discriminator depth is set to $L_{d}=2$, with the intermediate hidden layer width configured to 128, followed by a classification layer outputting the environmental predictions. The width of each layer is denoted as $D_{l}$, and the output is an environmental prediction distribution:

(15) $${\hat{e}}_{i}=\mathrm{Softmax}\left(W_{d}^{L_{d}}\phi \left(\cdots \phi \left(W_{d}^{1}q_{i}^{risk}+b_{d}^{1}\right)\right)+b_{d}^{L_{d}}\right).$$

The risk encoder is encouraged to make $q_{i}^{risk}$ difficult for environmental prediction, whereas the environmental discriminator attempts to correctly identify the environment. To technically execute this adversarial training within a standard automatic differentiation framework, a Gradient Reversal Layer is inserted between the causal gating layer and the environmental discriminator. During the forward propagation, this layer acts as an identity transformation, allowing the discriminator to learn environmental features. During the backward propagation, it multiplies the passing gradients by a negative scaling factor, reversing the optimization direction for the upstream encoder. Therefore, a minimax optimization is formed:

(16) $$\min_{\theta_{c},\theta_{y}}\max_{\theta_{d}}\;J=L_{y}\left(g\left(q_{i}^{risk}\right),y_{i}\right)-\alpha L_{e}\left(d\left(q_{i}^{risk}\right),e_{i}\right)+\beta {∥q_{i}^{risk⊤}q_{i}^{env}∥}_{F}^{2}.$$

where $L_{y}$ denotes the anomaly classification loss, $L_{e}$ denotes the environmental discrimination loss, and the last term enforces orthogonality between the risk component and the environmental component to reduce information overlap. The rationale can be explained as follows. If $q_{i}^{risk}$ still contains substantial environmental information, then a discriminator d(·) exists such that $L_{e}$ becomes small. In minimax training, the risk encoder and the environmental discriminator engage in an adversarial game that can be formally interpreted as minimizing a variational upper bound on the mutual information $I(q_{i}^{risk};e_{i})$. Let the discriminator $D_{\phi }\left(e_{i}|q_{i}^{risk}\right)$ serve as a variational approximation of the true posterior $P(e_{i}|q_{i}^{risk})$. According to the variational information bound framework, the mutual information is rigorously bounded by:

(17) $$I\left(q_{i}^{risk};e_{i}\right)=H\left(e_{i}\right)-H\left(e_{i}|q_{i}^{risk}\right)\leq H\left(e_{i}\right)+E_{q,e}\left[\log D_{\phi }\left(e_{i}|q_{i}^{risk}\right)\right].$$

During the adversarial optimization process, the discriminator maximizes the expected log-likelihood $E\left[\log D_{\phi }\left(e_{i}|q_{i}^{risk}\right)\right]$ to tighten this bound, while the risk encoder minimizes this term, which is mathematically equivalent to maximizing the adversarial loss $L_{e}$, to drive the upper bound of the mutual information toward zero. When the minimax game reaches equilibrium, the encoder completely obfuscates the environmental information, forcing the optimal variational posterior $D_{\phi }\left(e_{i}|q_{i}^{risk}\right)$ to degenerate to the marginal prior $P(e_{i})$. Under this condition, the formal constraint is achieved:

(18) $$\lim_{\mathrm{equilibrium}}I\left(q_{i}^{risk};e_{i}\right)\leq H\left(e_{i}\right)-H\left(e_{i}\right)=0.$$

Therefore, $q_{i}^{risk}$ is formally constrained to be independent of environmental variables, and the dependence of the model on pseudo-correlated factors such as regions, transportation modes, port distributions, and textual styles is mathematically weakened. Meanwhile, the classification loss still requires $q_{i}^{risk}$ to retain information that is discriminative for the anomaly label $y_{i}$. Consequently, a stable risk representation that is effective for the label but insensitive to the environment is learned. When applied to the proposed task, this module can prevent the model from incorrectly treating high-frequency routes, specific country attributes, fixed transportation modes, or common textual expressions as risk origins. Instead, the model is encouraged to focus on clues with engineering causal meanings, such as conflicts between commodity descriptions and HS codes, price deviations, inconsistencies in quantity and weight, abnormal logistics paths, abnormal inspection records, and abnormal coupling of entity relationships, thereby improving generalization ability and explanatory reliability across time, regions, ports, and entities.

The counterfactual perturbation generator is parameterized as a 2-layer feedforward network. It takes the concatenation of the 256-dimensional stable risk representation and a 16-dimensional learnable embedding corresponding to the specific intervention factor as input. The hidden layer dimension is configured to 128, and the output dimension aligns with the 256-dimensional latent space to facilitate direct representation addition. The shared risk evaluator is constructed as a 3-layer fully connected classification network, featuring progressively decreasing hidden dimensions of 128 and 64, terminating in a 1-dimensional output bounded by a Sigmoid activation function to yield the final anomaly risk probability. Consistent with the causal encoding network, Layer Normalization and Gaussian Error Linear Unit activations are applied throughout to maintain optimization stability. Furthermore, to guarantee that the generated counterfactual samples reflect realistic operational changes rather than unconstrained mathematical artifacts, the perturbation magnitude regularization weight is strictly set to 0.1. This restricts the latent counterfactual shifts to a bounded neighborhood around the original sample, ensuring that the theoretical response analysis remains practically grounded and fully reproducible.

#### 3.2.4. Counterfactual Anomaly Response Enhancement Module

The counterfactual-inference-based anomaly discrimination enhancement module receives the stable risk representation $r_{i}\in R^{C_{r}}$ after causal debiasing and constructs counterfactual risk responses around key intervenable factors in cross-border supervision. This module consists of a counterfactual perturbation generator, a shared risk evaluator, and a response consistency constraint.

**Figure 4:**
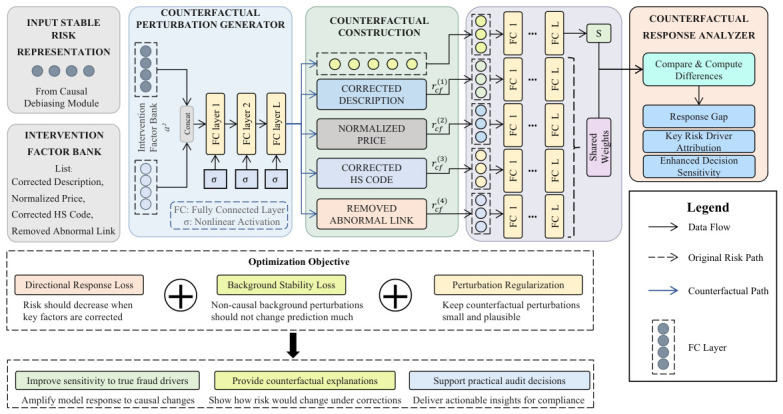
The counterfactual anomaly response enhancement module constructs intervention representations for key risk factors and compares risk response changes to improve the identification and explanation of true anomaly drivers.

As shown in Figure 4, the counterfactual perturbation generator adopts an $L_{f}$-layer feedforward network. The input width of each layer is $F_{l}$, the output width is $F_{l+1}$, the height in the sample dimension is *B*, and the number of channels corresponds to the latent risk variable dimension $F_{l}$. Its input is the concatenation of the stable risk representation $r_{i}$ and the intervenable field embedding $a_{i}^{k}$, where $a_{i}^{k}$ denotes the *k*-th key variable, such as commodity description standardization, HS code consistency, price deviation degree, declared weight consistency, logistics route deviation degree, or entity relationship abnormality. The counterfactual perturbation generation process can be expressed as:

(19) $$\delta_{i}^{k}=W_{f}^{L_{f}}\psi \left(W_{f}^{L_{f}-1}\cdots \psi \left(W_{f}^{1}\left[r_{i};a_{i}^{k}\right]+b_{f}^{1}\right)+b_{f}^{L_{f}-1}\right)+b_{f}^{L_{f}},$$

where $W_{f}^{l}\in R^{F_{l+1}\times F_{l}}$, $b_{f}^{l}\in R^{F_{l+1}}$, ψ(·) denotes the nonlinear activation function, and $\delta_{i}^{k}$ denotes the counterfactual perturbation generated in the latent space after intervention on the *k*-th type of risk factor. Compared with directly modifying original document fields or sensor records, generating counterfactual samples in the latent representation space can avoid the problems of text-field discreteness, non-differentiable categorical fields, difficulty in continuously modifying logistics trajectories, and difficulty in directly optimizing relationship links. The counterfactual risk representation is defined as:

(20) $${\bar{r}}_{i}^{k}=r_{i}+T_{k}\delta_{i}^{k},$$

where $T_{k}\in R^{C_{r}\times F_{L_{f}}}$ is the projection matrix corresponding to the *k*-th type of intervention variable and is used to map the counterfactual perturbation back to the risk representation space. Subsequently, the original representation $r_{i}$ and the counterfactual representation ${\bar{r}}_{i}^{k}$ share the same risk evaluator p(·), which is composed of an $L_{p}$-layer fully connected classification network. The width of each layer is denoted as $P_{l}$, and the final output is the anomaly risk probability:

(21) $$s_{i}=\sigma \left(p(r_{i};\theta_{p})\right),\;{\bar{s}}_{i}^{k}=\sigma \left(p({\bar{r}}_{i}^{k};\theta_{p})\right).$$

To make counterfactual changes consistent with cross-border supervision logic, directional constraints are imposed on different types of variables. If the *k*-th type of variable is corrected to a more reasonable state, such as standardized commodity descriptions, consistency between HS codes and commodity semantics, declared prices returning to normal intervals, consistency between declared weights and inspection weights, restored reasonable logistics routes, or removal of abnormal entity associations, the risk score of high-risk samples should decrease. This constraint can be expressed as:

(22) $$L_{dir}=\sum_{i}\sum_{k}\omega_{i}^{k}\max\left(0,\;{\bar{s}}_{i}^{k}-s_{i}+\gamma_{k}\right),$$

where $\omega_{i}^{k}$ denotes the weight indicating whether the *k*-th type of variable is intervenable for sample *i*, and $\gamma_{k}$ denotes the expected risk-change margin. For non-critical background variables, the model output should not change drastically under slight perturbations. Therefore, a stability constraint is further introduced:

(23) $$L_{sta}=\sum_{i}\sum_{k\in B}{∥p\left(r_{i};\theta_{p}\right)-p\left({\bar{r}}_{i}^{k};\theta_{p}\right)∥}_{2}^{2},$$

where B denotes the set of background variables. The final objective for counterfactual discrimination enhancement is:

(24) $$L_{cf}^{new}=\eta_{1}L_{dir}+\eta_{2}L_{sta}+\eta_{3}\sum_{i}\sum_{k}{∥\delta_{i}^{k}∥}_{2}^{2}.$$

where the last term is used to constrain the magnitude of counterfactual perturbations, ensuring that the generated counterfactual samples do not deviate excessively from the original samples. Its mathematical rationale can be explained through local risk response analysis. By performing a first-order expansion of the shared risk evaluator around $r_{i}$, the following can be obtained:

(25) $${\bar{s}}_{i}^{k}-s_{i}\approx \nabla_{r}p{\left(r_{i};\theta_{p}\right)}^{⊤}T_{k}\delta_{i}^{k}.$$

Therefore, if an intervention direction $T_{k}\delta_{i}^{k}$ is highly aligned with the risk gradient direction, the corresponding variable has a significant influence on model discrimination. If the two directions are approximately orthogonal, the corresponding variable contributes little to the risk output. By minimizing $L_{dir}$, the model is forced to produce a risk decrease consistent with logic when key risk variables are corrected. By minimizing $L_{sta}$, the model remains stable under perturbations of background variables. Accordingly, for the key risk set K and the background set B, the model learning objective approximately satisfies:

(26) $$\left|\nabla_{r}p{\left(r_{i};\theta_{p}\right)}^{⊤}T_{k}\delta_{i}^{k}\right|>\left|\nabla_{r}p{\left(r_{i};\theta_{p}\right)}^{⊤}T_{b}\delta_{i}^{b}\right|,\;k\in K,\;b\in B.$$

This indicates that the model becomes more sensitive to true risk factors near the decision boundary while remaining more robust to non-causal background factors. When applied to the proposed task, this module can extend anomaly detection from simple probability output to an intervenable risk explanation process. For example, when a declaration sample is classified as high risk, the changes in the risk score after standardizing commodity descriptions, correcting HS codes, adjusting declared prices, calibrating weight records, restoring reasonable logistics paths, or removing abnormal entity associations can be further analyzed, so that the most important anomaly sources can be identified.

To ensure that these latent-space counterfactuals reflect realistic operational changes, the intervention magnitude is rigorously justified and verified. Specifically, the magnitude constraint governed by the parameter $\eta_{3}$ is dynamically calibrated based on the statistical variance of the corresponding feature distribution observed in historical normal samples. This guarantees that the synthetic perturbations remain strictly bounded within the feasible operational ranges permitted by current customs regulations. Furthermore, to validate the practical plausibility of this module, qualitative assessments were systematically conducted by domain experts, including senior customs auditors. The experts reviewed concrete operational examples mapped from the latent interventions. For instance, in a scenario where a high risk score was primarily driven by a mismatch between the declared weight and the port weighbridge record, the latent counterfactual perturbation simulated the operational correction of calibrating the declared weight. The experts confirmed that the corresponding substantial decrease in the predicted risk score accurately mirrored real-world inspection logic, where successfully resolving such a discrepancy clears the regulatory suspicion. Similarly, latent shifts corresponding to normalizing an abnormally low declared price to a standard market interval, or amending an HS code to align perfectly with the semantic commodity description, were verified by the auditors as standard, logical regulatory rectification procedures. Consequently, these expert validations demonstrate that the generated counterfactuals robustly correspond to realistic inspection scenarios, thereby providing actionable and practically sound evidence for risk tracing and making the inference-stage results strictly consistent with practical regulatory requirements.

## 4. Results and Discussion

### 4.1. Experimental Configuration

#### 4.1.1. Hardware and Software Platform

The experiments were conducted on a high-performance hardware platform equipped with graphics processing units and multicore central processing units to support the training and inference of the proposed multimodal deep model. Specifically, the server was equipped with an NVIDIA A100 GPU based on the NVIDIA Ampere architecture, with 40 GB of video memory, which effectively supports large-scale text encoding and multimodal feature fusion. The central processing unit was an Intel Xeon Gold 6230, which provides multicore parallel computing capability for accelerating data preprocessing and batch loading. The system memory was configured as 128 GB to ensure stable operation for large-scale samples and intermediate feature caching. NVMe solid-state drives were used for storage to improve data reading and writing efficiency. The overall hardware environment satisfies the computational performance and storage bandwidth requirements of cross-border multisource heterogeneous data modeling and provides favorable stability and scalability during model training.

For the software platform, the experimental system was built on a mainstream deep learning development environment. Ubuntu 20.04 was adopted as the operating system to ensure compatibility and stability. PyTorch was selected as the core implementation framework for model construction, automatic differentiation, and training optimization. The text encoding component relied on pretrained language model interfaces provided by Hugging Face Transformers to support semantic representation learning for cross-border document texts. Graph-structured modeling was implemented using PyTorch Geometric to model enterprise relationships and trade-chain features. Data processing and analysis were conducted using NumPy and Pandas. The overall software environment supports complex procedures such as multimodal data processing, causal-constraint training, and counterfactual sample generation, thereby providing a reliable basis for the experiments.

In the experimental setting, to rigorously prevent any potential temporal data leakage and resolve any ambiguity regarding the data partitioning, the combination of the fixed split and cross-validation is clearly defined. The evaluation does not rely on two conflicting procedures; instead, the 7:1:2 ratio is dynamically integrated into an out-of-time 5-fold cross-validation strategy applied across the entire dataset. Specifically, the data sequence was divided into chronological blocks using a rolling-window approach. Within each of the 5 folds, the corresponding historical data window was partitioned into a training set and a validation set, while the strictly subsequent future block served as the test set, maintaining an exact overall sample ratio of 7:1:2 for training, validation, and testing along the timeline. This ensures that in every single fold, all test samples strictly occurred after the training and validation periods. The averaged results over these multiple rolling runs were taken as the final performance indicators. Furthermore, we implemented strict data isolation strategies during feature engineering. It is guaranteed that enterprise relationship features and historical declaration statistics were computed exclusively using past records available prior to the exact timestamp of each target sample. No future information was exposed during the construction of entity relationship graphs or historical statistical fields, and samples from the same enterprise were strictly constrained by the timeline to prevent future data from leaking into the training phase, thereby ensuring that the reported performance reflects genuine predictive capability and realistic cross-region generalization.

During model training, the key hyperparameters were set as follows. The maximum sequence length of the text encoder was set to 256 to balance semantic representation capability and computational cost. The overall model was trained with a batch size of 32. AdamW was selected as the optimizer, with the initial learning rate set to $\alpha =2\times 10^{-5}$, and a linear warm-up and decay strategy was adopted to improve training stability. The hidden dimension of the multimodal fusion layer was set to 256 to ensure effective representation of different modality features in a unified space. The weighting coefficients of the causal constraint loss and the counterfactual loss were set to $\lambda_{1}=0.5$ and $\lambda_{2}=0.3$, respectively, to balance classification performance and causal robustness. The number of training epochs was set between 20 and 30, and an early-stopping strategy based on validation-set performance was adopted to prevent overfitting. In addition, during counterfactual sample construction, the perturbation magnitude of key fields was controlled within a reasonable range to ensure that the generated samples were both interpretable and consistent with logic. The overall hyperparameter configuration was obtained through multiple rounds of tuning, achieving a favorable balance between performance and stability.

#### 4.1.2. Baseline Models and Evaluation Metrics

In the comparative experiments, several representative models were selected for performance comparison, including traditional machine learning models such as Logistic Regression [36], Random Forest [37], and XGBoost [38]; deep learning models using only structured features, such as MLP [39] and TabTransformer [40]; models using only the textual modality, such as TextCNN [41], BiLSTM [42], and BERT [43]; conventional multimodal fusion models, such as BERT+MLP [43] and Multimodal Transformer [9]; and recent state-of-the-art advanced frameworks, including the graph neural network model MSDG [44], the multimodal generative framework MFGAN [45], the causality-aware domain generalization method CSRDN [46], and the multi-modal domain generalization approach MDGAR [47]. Logistic Regression is a classical classification model based on a linear assumption, in which the relationship between features and labels is characterized by parameters β. Its advantage lies in its simple structure and favorable interpretability. Random Forest performs prediction by constructing multiple decision trees and conducting ensemble learning, thereby effectively reducing the risk of overfitting, with strong capability in modeling nonlinear relationships. XGBoost is an ensemble model based on the gradient boosting framework, in which high-accuracy prediction is achieved through iterative optimization of the loss function L; it is characterized by stable performance and excellent results on structured data. MLP, as a multilayer feedforward neural network, learns complex feature representations through multilayer nonlinear mappings and is advantageous due to its flexible structure and ease of implementation. TabTransformer introduces a self-attention mechanism to model tabular data, allowing relationships among features to be adaptively learned through attention weights α, thereby capturing feature interactions. TextCNN extracts local semantic features through convolutional operations and is suitable for short-text modeling, with high computational efficiency and sensitivity to local patterns. BiLSTM captures contextual dependencies through bidirectional sequence modeling and can learn sequential features, showing strong capability in contextual semantic modeling. BERT is pretrained based on the Transformer architecture and obtains deep semantic representations through context-aware encoding, providing strong language understanding ability. BERT+MLP is a simple multimodal fusion method in which textual representations and structured features are concatenated before being fed into a classifier, with the advantages of simple implementation and stable fusion performance. Multimodal Transformer realizes information interaction among different modalities through cross-modal attention mechanisms, dynamically fusing representations through attention weights α and effectively modeling complex associations among multiple modalities. MSDG captures complex topological associations among entities through a multi-scale dynamic graph neural network. MFGAN utilizes an attention-based autoencoder and a generative adversarial network to achieve robust multimodal fusion. CSRDN leverages domain intervention from a causality-aware perspective to learn invariant features for domain generalization. MDGAR incorporates a multi-modal approach specifically targeting domain generalization to enhance robustness across different environments.

For model evaluation, Accuracy was used to measure the overall classification correctness, Precision was used to characterize the proportion of truly positive samples among samples predicted as positive, Recall was used to measure the ability to identify truly positive samples, F1-score was used to balance Precision and Recall, AUC was used to evaluate the overall ranking ability of the model, PR-AUC was used to emphasize positive-class identification under class imbalance, Top-K Hit Rate was used to measure whether high-risk samples appeared among the top *K* predicted results, and Early Warning Gain was used to evaluate the warning benefit of the model under limited screening resources.

(27) $$\mathrm{Accuracy}=\frac{TP+TN}{TP+TN+FP+FN},$$

(28) $$\mathrm{Precision}=\frac{TP}{TP+FP},$$

(29) $$\mathrm{Recall}=\frac{TP}{TP+FN},$$

(30) $$F1=\frac{2\cdot \mathrm{Precision}\cdot \mathrm{Recall}}{\mathrm{Precision}+\mathrm{Recall}},$$

(31) $$\mathrm{AUC}=\int_{0}^{1}TPR\left(FPR^{-1}\left(x\right)\right)dx,$$

(32) $$\text{PR-AUC}=\int_{0}^{1}\mathrm{Precision}\left(Recall\right)dRecall,$$

(33) $$\text{Top-K Hit Rate}=\frac{\mathrm{Number}\;\mathrm{of}\;\mathrm{positives}\;\mathrm{in}\;\mathrm{Top-K}}{\text{Total}\;\mathrm{positives}},$$

(34) $$\mathrm{Early}\;\mathrm{Warning}\;\mathrm{Gain}=\frac{\mathrm{Captured}\;\mathrm{risk}\;\mathrm{in}\;\text{Top-K}}{\mathrm{Total}\;\mathrm{risk}}.$$

In the above equations, TP denotes true positives, TN denotes true negatives, FP denotes false positives, and FN denotes false negatives. TPR denotes the true positive rate, namely Recall, FPR denotes the false positive rate, and *K* denotes the sample-number threshold for screening. “Captured risk” denotes the number of risk samples captured among the top *K* samples, while “Total risk” denotes the total number of risk samples in the dataset.

### 4.2. Performance Comparison with Baseline Models

This experiment was designed to evaluate the overall effectiveness of the proposed method in cross-border logistics anomaly monitoring and to systematically compare it with traditional machine learning methods, single-modality deep learning models, and conventional multimodal fusion models. To ensure the statistical reliability of our findings, all experimental results are reported as the mean ± standard deviation across the 5-fold cross-validation. Furthermore, we conducted a paired *t*-test to assess the statistical significance of the performance improvements. The specific *p*-values comparing the proposed method against the best-performing baseline for each metric are explicitly reported in the bottom row of the table, confirming that the improvements are statistically significant (e.g., p=0.014 for Accuracy and p=0.011 for F1-score).

**Figure 5:**
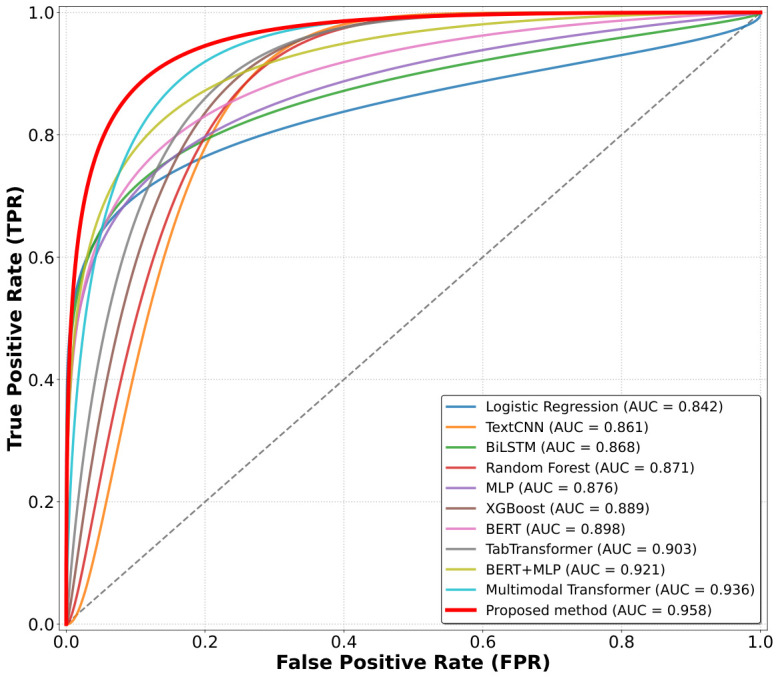
ROC curve comparison of different models for cross-border fraud detection, where the proposed method achieves the highest AUC and demonstrates superior risk identification performance.

As shown in Table 2 and Figure 5, Logistic Regression achieved Accuracy, F1-score, and AUC values of 0.821, 0.646, and 0.842, respectively, showing the weakest overall performance. This indicates that a linear model is insufficient for characterizing the complex nonlinear risk relationships in cross-border data. Random Forest and XGBoost increased the F1-score to 0.684 and 0.707, respectively, suggesting that ensemble tree models are better able to handle nonlinear combinations in structured declaration fields. However, their utilization of textual semantics, logistics states, and entity relationships remains limited. The F1-score of MLP was 0.691, which was slightly higher than that of Random Forest but lower than that of XGBoost. This indicates that multilayer nonlinear mapping provides a certain level of representation capability, but heterogeneous signals cannot be sufficiently modeled without an explicit feature interaction mechanism. TabTransformer achieved an F1-score and AUC of 0.728 and 0.903, respectively, outperforming conventional structured-data models. This demonstrates that the self-attention mechanism can more effectively capture dependencies among fields such as price, weight, HS code.

From the results of textual and multimodal models, the F1-scores of TextCNN and BiLSTM were 0.669 and 0.677, respectively. These models can extract local semantic patterns or contextual dependencies, but they remain insufficient for modeling complex long-range semantics and multisource signal interactions. The F1-score of BERT increased to 0.722, indicating that pretrained language models can better understand deep semantics in commodity descriptions, bill-of-lading notes, and contract summaries. BERT+MLP achieved an F1-score of 0.758, showing that simple concatenation of textual semantics and structured fields can already produce clear performance gains. Multimodal Transformer further achieved an Accuracy of 0.903 and an F1-score of 0.783, indicating that cross-modal attention can dynamically model dependencies among different modalities. To further validate against recent state-of-the-art advances, the newly added baselines were evaluated. MSDG achieved an F1-score of 0.793 and an AUC of 0.941 by leveraging graph structures to model complex entity relationships. MFGAN reached an F1-score of 0.801, demonstrating the effectiveness of generative adversarial networks in multimodal feature fusion. Furthermore, the domain-generalization methods CSRDN and MDGAR achieved F1-scores of 0.806 and 0.813, respectively. Their strong performance, along with high AUC values of 0.948 and 0.951, highlights that incorporating causal constraints and domain-invariant learning significantly improves model robustness. Despite the highly competitive performance of these advanced frameworks, the proposed method achieved the best results across all metrics, with Accuracy, Precision, Recall, F1-score, AUC, and PR-AUC reaching 0.927, 0.842, 0.811, 0.826, 0.958, and 0.817, respectively. This improvement is mainly attributed to the fact that multisource sensing signal fusion is combined with causal debiasing to weaken the influence of pseudo-correlated factors such as region, transportation mode, and textual style, while counterfactual responses are used to enhance model sensitivity to true anomaly-driving factors. Therefore, compared with models that only learn statistical correlations, the proposed method can obtain a more stable, explainable, and cross-scenario-adaptive risk representation.

### 4.3. Generalization and Robustness Evaluation

This experiment was designed to evaluate the generalization ability and robustness of the proposed method in realistic cross-border supervision environments, rather than merely assessing static classification performance under a random test split. All results in these evaluation scenarios are presented as the mean ± standard deviation to accurately reflect the model variance across different folds and perturbation settings.

**Figure 6:**
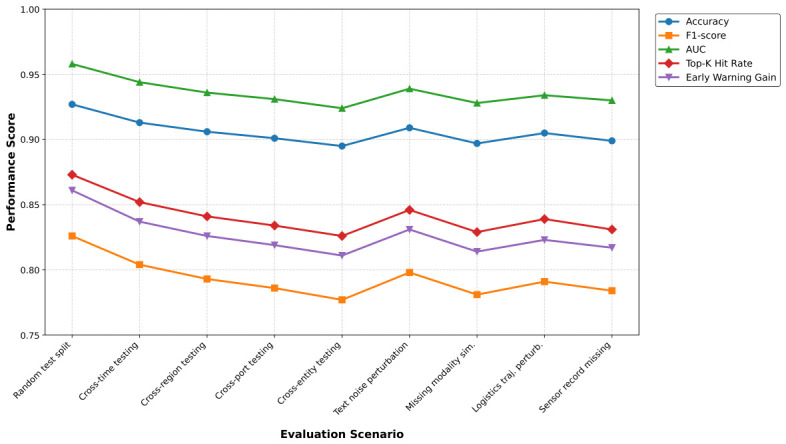
Performance comparison across different evaluation scenarios shows that the proposed method maintains stable detection performance under random split, cross-time, cross-region, cross-port, cross-entity, and multiple perturbation settings.

As shown in Table 3 and Figure 6, the random test split represents the standard testing scenario, under which the model achieved an Accuracy of 0.927, an F1-score of 0.826, and an AUC of 0.958. This indicates that when the training and test distributions are relatively consistent, the proposed method can sufficiently exploit multisource sensing signals to complete anomaly identification. In cross-time testing, Accuracy and F1-score decreased to 0.913 and 0.804, respectively, suggesting that model performance is affected when commodity prices, transportation routes, and anomaly strategies change over time. However, the performance degradation remained limited, indicating that the causal debiasing mechanism can alleviate pseudo-correlation interference caused by temporal drift. The F1-scores of cross-region and cross-port testing were 0.793 and 0.786, respectively, showing that differences in commodity structures, regulatory procedures, logistics nodes, and declaration habits across regions and ports lead to more obvious distribution shifts. Nevertheless, the model maintained a high AUC, demonstrating stable risk-ranking capability. In cross-entity testing, the F1-score decreased to 0.777, which represents a relatively large drop among the generalization scenarios. This suggests that historical behavior patterns, transaction relationships, and declaration habits of unseen enterprise entities vary substantially, imposing higher requirements on the model.

From the robustness results, the model still achieved an Accuracy of 0.909 and an F1-score of 0.798 under text noise perturbation, which was quantitatively defined by randomly replacing or masking 15% of the input tokens. This indicates that the pretrained language encoder and cross-modal fusion mechanism can resist OCR errors, abbreviation confusion, and nonstandard commodity descriptions to a certain extent. Under missing modality simulation, where structured or relational features were randomly dropped for 20% of the samples, the F1-score was 0.781, showing that model performance declines when partial document, logistics, or relationship signals are unavailable. However, the gated fusion structure can dynamically adjust information contributions according to the available modalities, preventing severe degradation. Under logistics trajectory perturbation, generated by adding Gaussian noise with a standard deviation of 0.01 degrees to the longitude and latitude coordinates, and sensor record missing scenarios, where 15% of hardware records were randomly set to null, the F1-scores were 0.791 and 0.784, respectively. These results indicate that hardware sensing signals such as GPS, AIS, weighing, and electronic seals make important contributions to risk identification, while the model does not completely rely on a single sensing source. Theoretically, the stability of the proposed method comes from three aspects. Cross-modal attention establishes complementary relationships among different signals and prevents noise from a single input source from dominating the decision. Causal representation learning suppresses unstable correlations in regional, port, and entity backgrounds, making the risk representation closer to the anomaly mechanisms shared across scenarios. Counterfactual response constraints enhance model sensitivity to changes in key risk variables. Therefore, even when time, region, entity, and sensing-signal quality change, the model can still maintain strong risk-ranking and priority-screening capabilities.

### 4.4. Ablation Study

This ablation experiment was designed to verify the independent contribution of each component in the proposed multisource sensing fusion and causally explainable framework, and to analyze the effects of different modalities, alignment mechanisms, causal debiasing, counterfactual response, and engineering constraints on final anomaly identification performance. Similar to the baseline comparisons, the results are presented as the mean ± standard deviation. Paired *t*-tests were conducted to compare the full model against each ablation variant. The specific *p*-values, demonstrating the statistical significance of the performance drop when removing a core module compared to the best-performing variant, are explicitly added to the bottom row of the table.

**Figure 7:**
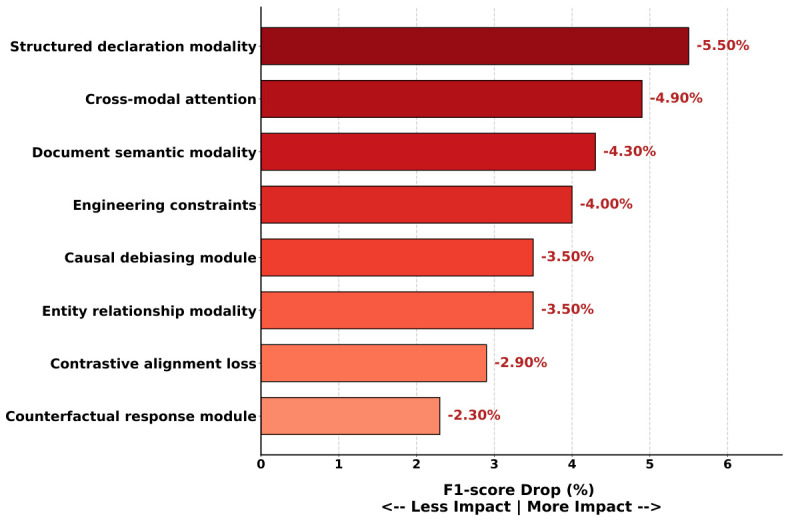
The F1-score drop comparison after module ablation shows that the structured declaration modality, cross-modal attention, and document semantic modality contribute most significantly to model performance.

As shown in Table 4 and Figure 7, the full model achieved the best results across all metrics, with Accuracy, Precision, Recall, F1-score, AUC, and PR-AUC reaching 0.927, 0.842, 0.811, 0.826, 0.958, and 0.817, respectively. This indicates that multisource signal fusion, causal constraints, and counterfactual explanation form an effective complementary relationship. After the document semantic modality was removed, the F1-score decreased to 0.783, indicating that semantic information contained in commodity descriptions, bill-of-lading notes, and contract summaries is important for identifying ambiguous commodity names, inconsistent descriptions, and implicit anomalies. After the structured declaration modality was removed, the F1-score further dropped to 0.771, which was the largest decrease among the single-modality removal experiments. This suggests that fields such as price, weight, quantity, HS code are fundamental discriminative signals for cross-border anomaly monitoring. After the entity relationship modality was removed, the F1-score was 0.791. Although the decline was smaller than those caused by removing the first two modalities, the result was still clearly lower than that of the full model, indicating that enterprise historical cooperation, upstream and downstream associations, and risk co-occurrence information can supplement group-level anomaly patterns that are difficult to capture from a single declaration sample. When only document and structured fields were used, the F1-score decreased to 0.758, and when only structured fields were used, it further decreased to 0.728, demonstrating that single-modality or simple dual-modality inputs cannot sufficiently cover the multisource risk mechanisms in real cross-border scenarios.

From the perspective of model structure, removing cross-modal attention reduced the F1-score to 0.777, indicating that simple concatenation cannot effectively establish fine-grained correspondences among document semantics, declaration fields, and entity relationships, such as the implicit consistency among commodity descriptions and HS codes, declared weights and commodity attributes, and logistics routes. Removing the contrastive alignment loss resulted in an F1-score of 0.797, showing that cross-modal contrastive learning can enhance latent-space consistency among different sensing signals from the same sample and reduce semantic fragmentation across modalities. After the causal debiasing module was removed, the F1-score decreased to 0.791, suggesting that if only statistical correlations are learned, the model is more likely to be affected by pseudo-correlated factors such as regions, ports, transportation modes, and textual styles, resulting in insufficiently stable risk representations. Removing the counterfactual response module led to an F1-score of 0.803, indicating that counterfactual perturbations can enhance model sensitivity to key intervenable factors and guide the model to focus more on variables that truly drive risk changes. After engineering constraints were removed, the F1-score decreased to 0.786, demonstrating that business constraints such as consistency between commodity descriptions and HS codes, matching between declared and inspected weights, and reasonableness of logistics paths provide more stable structural priors for the model. Overall, the full model achieves the best performance because its mathematical modeling process does not merely expand the feature dimensionality. Instead, cross-modal attention is used to learn dependencies among heterogeneous signals, contrastive constraints are used to reduce representation distances among signals from the same sample, causal debiasing is used to reduce the interference of environmental variables in risk representations, and counterfactual responses are used to strengthen decision boundaries along key risk directions. Therefore, simultaneous advantages are achieved in accuracy, recall capability, and imbalanced-risk identification.

### 4.5. Hyperparameter Sensitivity Analysis

To evaluate the robustness of the chosen configuration and to systematically investigate the impact of key hyperparameters on model performance, a comprehensive hyperparameter sensitivity analysis was conducted. This experiment aims to justify the selected values by observing the fluctuations in the final prediction metrics when individual hyperparameters are varied within a reasonable range. The analysis focuses on four core parameters: batch size, learning rate α, causal constraint loss weight $\lambda_{1}$, and counterfactual loss weight $\lambda_{2}$. During the evaluation, one target hyperparameter was adjusted while the others were kept at their optimal baseline settings. The F1-score and AUC metrics derived from the 5-fold cross-validation were recorded to measure the model sensitivity.

As shown in Table 5, the proposed framework demonstrates a high degree of robustness across different hyperparameter configurations, with F1-score and AUC values remaining relatively stable within the tested ranges. For the batch size, performance peaks at 32, balancing gradient stability and adequate noise for generalization, whereas a larger batch size of 64 slightly degrades the F1-score to 0.819. Regarding the learning rate, the model is somewhat sensitive; an optimal value of $2\times 10^{-5}$ effectively prevents underfitting observed at $1\times 10^{-5}$ and optimization divergence observed at $5\times 10^{-5}$. The loss weights $\lambda_{1}$ and $\lambda_{2}$ control the contribution of the causal debiasing and counterfactual response modules, respectively. When $\lambda_{1}$ is set to 0.5 and $\lambda_{2}$ to 0.3, the model achieves the best balance between accurate anomaly classification and robust causal representation. Deviating from these optimal values results in only minor performance drops, indicating that the framework does not excessively rely on rigorous hyperparameter tuning to achieve state-of-the-art capability. These results systematically justify the parameter choices reported in the experimental setup.

### 4.6. Discussion

The results indicate that cross-border logistics and financial anomaly monitoring cannot rely solely on a single declaration field or document text. Instead, the entire cargo and trade-finance circulation process should be considered, and multisource information such as electronic documents, declaration fields, logistics trajectories, port inspections, cold-chain environments, and entity relationships should be comprehensively utilized [48]. In practical port supervision scenarios, a batch of goods may contain a seemingly standardized commodity name in the declaration form, while subtle inconsistencies exist between the HS code and the commodity description. At the same time, the declared price may be lower than the historical interval of similar commodities, and the logistics trajectory may show abnormal transshipment [49]. If only manual rules or a single structured model are used, such anomalies may be fragmented into several insignificant local signals [50]. In contrast, the proposed method jointly models document semantics, declared prices, transportation routes, and entity relationships, making it easier to identify financial risks hidden in the coupling relationships among multisource signals. Especially in scenarios such as port container clearance, batch review of cross-border e-commerce parcels, agricultural product cold-chain export, and bonded warehouse entry and exit supervision, this method can provide regulators with more targeted risk-ranking results, enabling limited inspection resources to be prioritized toward more suspicious cargo batches.

From the perspective of practical value, the proposed method focuses not only on whether an anomaly exists, but also on where the anomaly may originate. For example, in agricultural product cold-chain export scenarios, if the declaration information for a batch of fruit or aquatic products is relatively complete, but temperature and humidity sensor records indicate a prolonged temperature abnormality during transportation and electronic seal records indicate unplanned container opening, the model can jointly incorporate environmental-state anomalies and logistics-node anomalies into the decision, thereby providing evidence for cargo-quality risk assessment, document-consistency verification, and financial compliance review [51]. In port clearance scenarios, if the weighbridge result deviates substantially from the declared weight, the X-ray image indicates inconsistency between loading density and the declared commodity type, and the enterprise historical transaction relationship shows an abnormally high-frequency cooperation chain, the model can jointly explain weight anomalies, inspection-image anomalies, and entity-relationship anomalies as sources of high risk [52]. This explanation pattern is closer to the practical working logic of regulators, because port inspection usually does not rely on a single indicator for direct determination, but instead requires a comprehensive judgment based on documents, goods, routes, device records, and enterprise backgrounds.

In addition, the cross-time, cross-region, cross-port, and cross-entity testing results demonstrate that the proposed method maintains relatively stable performance under changing environments, which is important for real deployment. Commodity prices, transportation routes, enterprise cooperation relationships, and declaration habits in cross-border trade continuously change with seasons, markets, and policies. If a model only learns superficial correlations in the training data, it may easily fail when applied to new ports, new enterprises, or new routes. Through causal debiasing and engineering constraints, the proposed method focuses more on stable rules, such as consistency between commodity descriptions and HS codes, rationality of declared prices, matching between weight and inspection records, continuity of logistics paths, and abnormal coupling of entity relationships. Therefore, it is more suitable for continuously changing regulatory and financial security environments. For regulatory departments, this method can be embedded into existing workflows as an intelligent auxiliary screening tool, providing risk priorities, anomaly-source prompts, and key inspection suggestions before manual review, thereby improving port clearance efficiency and financial anomaly identification accuracy.

### 4.7. Limitations and Future Work

Although a multisource sensing signal fusion framework for cross-border logistics anomaly monitoring was constructed and its effectiveness was verified under multiple experimental scenarios, certain limitations remain. First, although the data used in this study cover multiple types of information, including electronic documents, structured declaration fields, logistics trajectories, port inspection records, and cold-chain environmental states, data sources in real cross-border scenarios are more complex. Data standards are not fully consistent across different ports, enterprises, and logistics platforms, and some sensor records may suffer from missing values, delays, or inconsistent acquisition frequencies. Second, although causal debiasing and counterfactual response mechanisms were introduced, counterfactual samples were mainly generated in the latent representation space. Therefore, the reliability of the explanation results still needs to be further validated using regulatory expert knowledge and real intervention cases. In addition, hardware sensing data such as X-ray images, video records, and electronic seals have different coverage levels across scenarios, and model stability under extreme modality-missing conditions still needs to be further improved. Future work will be advanced in three directions. First, data sources will be further expanded by incorporating data from more ports, comprehensive bonded zones, cross-border e-commerce platforms, and cold-chain logistics enterprises, so as to improve the adaptability of the model to different scenarios. Second, fine-grained modeling of multisource hardware sensing data will be strengthened. For example, X-ray image features, video temporal behaviors, electronic seal opening and closing sequences, and continuous changes in cold-chain environments will be further fused, enabling the model to more accurately perceive cargo physical states and logistics process anomalies. Third, lightweight deployment schemes for practical regulatory systems will be explored to reduce inference latency and computational resource consumption. Expert feedback mechanisms will also be incorporated to continuously calibrate model explanation results, thereby promoting the practical application of the proposed method in intelligent port supervision, cross-border logistics security, and supply chain risk early warning.

## 5. Conclusions

To meet the requirements of multisource heterogeneous data perception and financial anomaly risk identification in cross-border circulation processes, a multisource sensing signal fusion and causally explainable risk identification framework is proposed. In this framework, electronic documents, structured declaration fields, GPS/AIS logistics trajectories, port weighing records, RFID data, electronic seal status, X-ray inspection images, cold-chain temperature and humidity records, and transportation vibration records are uniformly modeled as multisource sensing signals in cross-border trade scenarios. Accordingly, the traditional document and financial compliance review problem is extended into an engineering-oriented anomaly monitoring task for intelligent port supervision. In terms of methodological design, a multisource cross-border sensing signal representation and cross-modal alignment module is constructed to model the interactions among textual semantics, attribute fields, logistics status, device records, and entity relationships. Furthermore, an engineering-constraint-guided causal risk representation mechanism is introduced to reduce the interference of spurious correlated factors, such as regions, ports, transportation modes, and textual styles, in model judgments. Meanwhile, a counterfactual anomaly response module is constructed to enhance model sensitivity and interpretability with respect to true anomaly-driving factors through intervention analysis of key variables.

Experimental results show that the proposed method achieves the best overall performance in the cross-border financial anomaly detection task, with Accuracy, Precision, Recall, F1-score, AUC, and PR-AUC reaching 0.927, 0.842, 0.811, 0.826, 0.958, and 0.817, respectively, clearly outperforming baseline models including Logistic Regression, Random Forest, XGBoost, BERT, BERT+MLP, and Multimodal Transformer. In cross-time, cross-region, cross-port, and cross-entity testing scenarios, high F1-score and AUC values are still maintained, indicating favorable cross-scenario generalization capability. Under complex conditions such as text noise, missing modalities, logistics trajectory perturbations, and missing sensing records, only limited performance degradation is observed, verifying the robustness of the proposed method against incomplete sensing data and noise interference in real-world scenarios. Ablation experiments further demonstrate that cross-modal attention, contrastive alignment, causal debiasing, counterfactual response, and engineering constraints all make important contributions to performance improvement. Overall, a feasible and explainable technical pathway is provided for AI-driven multisource sensing data fusion, intelligent port anomaly monitoring, and supply-chain financial risk early warning in cross-border scenarios.

Despite the promising results, this study has several limitations that should be acknowledged and addressed in future work. First, the proposed framework relies heavily on supervised learning using historical labeled anomalies, which intrinsically constrains its ability to discover entirely novel, unknown trade-finance fraud patterns without prior annotation. Second, while the constructed dataset is extensive, it remains constrained to specific regions and cooperative enterprises, and the lack of evaluation against standardized external benchmarks limits the broader comparative assessment of the framework. Furthermore, the current evaluations are primarily conducted in an offline environment, meaning the absence of real-world online testing and deployment-level validation leaves potential challenges related to real-time processing latency and continuous model updating unexplored. Future research will focus on integrating unsupervised or semi-supervised anomaly discovery mechanisms, evaluating the framework on broader open-source benchmarks, and deploying the system in live port supervision environments to validate its real-time operational reliability.

## Figures and Tables

**Table 1:** Summary of multisource cross-border data and hardware sensors.

Data Name	Data Volume	Sensor Type
Electronic documents and commodity texts	128,460	Scanner/OCR device
Structured declaration fields	86,320	Port terminal/Declaration terminal
GPS land trajectories (Total)	42,680	GPS sensor
Proprietary operational GPS data	34,144	GPS sensor
Public GPS data	8536	GPS sensor
AIS maritime trajectories (Total)	31,900	AIS device
Proprietary operational AIS data	19,140	AIS device
Public AIS data	12,760	AIS device
Port weighing data	39,760	Weighbridge/Dynamic weighing sensor
X-ray inspection images	18,240	X-ray inspection device
RFID identification data	28,450	RFID reader/RFID tag
Electronic seal status data	24,630	Electronic seal/Door sensor
Cold-chain temperature and humidity data	35,720	Temperature/Humidity sensor
Transportation vibration data	16,850	Acceleration/Vibration sensor
Port video and image data	21,360	HD camera/LPR camera
Anomaly risk labels (Total)	86,320	Inspection/Review terminal
Positive anomaly samples	7520	Inspection/Review terminal
Negative normal samples	78,800	Inspection/Review terminal

**Table 2:** Performance comparison with baseline models on cross-border logistics anomaly monitoring.

Model	Accuracy	Precision	Recall	F1-Score	AUC	PR-AUC
Logistic Regression	0.821±0.006	0.684±0.007	0.612±0.008	0.646±0.007	0.842±0.005	0.631±0.008
Random Forest	0.846±0.005	0.713±0.006	0.657±0.007	0.684±0.006	0.871±0.005	0.669±0.006
XGBoost	0.862±0.004	0.736±0.005	0.681±0.006	0.707±0.005	0.889±0.004	0.694±0.005
MLP	0.851±0.005	0.721±0.006	0.663±0.007	0.691±0.006	0.876±0.005	0.681±0.006
TabTransformer	0.873±0.004	0.754±0.005	0.704±0.006	0.728±0.005	0.903±0.004	0.718±0.005
TextCNN	0.835±0.006	0.701±0.007	0.639±0.008	0.669±0.007	0.861±0.005	0.654±0.007
BiLSTM	0.842±0.005	0.708±0.006	0.648±0.007	0.677±0.006	0.868±0.005	0.662±0.006
BERT	0.867±0.004	0.748±0.005	0.697±0.006	0.722±0.005	0.898±0.004	0.711±0.005
BERT+MLP	0.889±0.004	0.781±0.005	0.736±0.006	0.758±0.005	0.921±0.003	0.746±0.005
Multimodal Transformer	0.903±0.004	0.806±0.004	0.762±0.005	0.783±0.004	0.936±0.003	0.771±0.004
MSDG	0.908±0.004	0.812±0.004	0.775±0.005	0.793±0.004	0.941±0.003	0.782±0.004
MFGAN	0.912±0.003	0.818±0.004	0.784±0.005	0.801±0.004	0.945±0.003	0.791±0.004
CSRDN	0.915±0.003	0.825±0.004	0.789±0.004	0.806±0.004	0.948±0.003	0.796±0.004
MDGAR	0.918±0.003	0.831±0.004	0.796±0.004	0.813±0.004	0.951±0.003	0.804±0.004
Proposed method	0.927±0.003	0.842±0.004	0.811±0.005	0.826±0.004	0.958±0.002	0.817±0.004
*p*-value (vs. best baseline)	0.014	0.021	0.008	0.011	0.003	0.015

**Table 3:** Generalization and robustness evaluation under different cross-border monitoring scenarios.

Evaluation Scenario	Accuracy	F1-Score	AUC	Top-K Hit Rate	Early Warning Gain
Random test split	0.927±0.003	0.826±0.004	0.958±0.002	0.873±0.004	0.861±0.005
Cross-time testing	0.913±0.005	0.804±0.006	0.944±0.004	0.852±0.005	0.837±0.006
Cross-region testing	0.906±0.006	0.793±0.007	0.936±0.005	0.841±0.006	0.826±0.007
Cross-port testing	0.901±0.006	0.786±0.007	0.931±0.005	0.834±0.006	0.819±0.007
Cross-entity testing	0.895±0.007	0.777±0.008	0.924±0.006	0.826±0.007	0.811±0.008
Text noise perturbation	0.909±0.005	0.798±0.006	0.939±0.004	0.846±0.005	0.831±0.006
Missing modality simulation	0.897±0.006	0.781±0.007	0.928±0.005	0.829±0.006	0.814±0.007
Logistics trajectory perturbation	0.905±0.006	0.791±0.007	0.934±0.005	0.839±0.006	0.823±0.007
Sensor record missing	0.899±0.006	0.784±0.007	0.930±0.005	0.831±0.006	0.817±0.007

**Table 4:** Ablation study of the proposed multisource sensing fusion and causal explanation framework.

Model Variant	Accuracy	Precision	Recall	F1-Score	AUC	PR-AUC
Full model	0.927±0.003	0.842±0.004	0.811±0.005	0.826±0.004	0.958±0.002	0.817±0.004
w/o document semantic modality	0.902±0.005	0.803±0.006	0.764±0.007	0.783±0.006	0.934±0.004	0.772±0.005
w/o structured declaration modality	0.894±0.006	0.791±0.007	0.752±0.008	0.771±0.007	0.926±0.005	0.759±0.007
w/o entity relationship modality	0.908±0.005	0.811±0.006	0.772±0.007	0.791±0.006	0.941±0.004	0.781±0.006
w/o cross-modal attention	0.899±0.005	0.798±0.006	0.758±0.007	0.777±0.006	0.931±0.005	0.766±0.006
w/o contrastive alignment loss	0.910±0.004	0.816±0.005	0.779±0.006	0.797±0.005	0.943±0.004	0.786±0.005
w/o causal debiasing module	0.907±0.005	0.809±0.006	0.773±0.007	0.791±0.006	0.939±0.004	0.779±0.006
w/o counterfactual response module	0.914±0.004	0.821±0.005	0.786±0.006	0.803±0.005	0.946±0.003	0.792±0.005
w/o engineering constraints	0.904±0.005	0.806±0.006	0.767±0.007	0.786±0.006	0.936±0.004	0.775±0.006
Only document + structured fields	0.889±0.006	0.781±0.007	0.736±0.008	0.758±0.007	0.921±0.005	0.746±0.007
Only structured fields	0.873±0.007	0.754±0.008	0.704±0.009	0.728±0.008	0.903±0.006	0.718±0.008
*p*-value (vs. best variant)	0.012	0.018	0.009	0.015	0.004	0.017

**Table 5:** Hyperparameter sensitivity analysis of the proposed framework.

Hyperparameter	Evaluated Value	F1-Score	AUC
Batch size	16	0.812±0.005	0.945±0.004
Batch size	32	0.826±0.004	0.958±0.002
Batch size	64	0.819±0.006	0.952±0.005
Learning rate α	$1\times 10^{-5}$	0.815±0.006	0.949±0.004
Learning rate α	$2\times 10^{-5}$	0.826±0.004	0.958±0.002
Learning rate α	$5\times 10^{-5}$	0.808±0.007	0.941±0.006
Causal loss weight $\lambda_{1}$	0.3	0.817±0.005	0.950±0.004
Causal loss weight $\lambda_{1}$	0.5	0.826±0.004	0.958±0.002
Causal loss weight $\lambda_{1}$	0.7	0.821±0.005	0.955±0.003
Counterfactual loss weight $\lambda_{2}$	0.1	0.814±0.006	0.946±0.005
Counterfactual loss weight $\lambda_{2}$	0.3	0.826±0.004	0.958±0.002
Counterfactual loss weight $\lambda_{2}$	0.5	0.819±0.005	0.951±0.004

## Data Availability

The original contributions presented in this study are included in the article. Further inquiries can be directed to the corresponding author.
